# Incidence and Risk Factors of Low Back Pain in Marathon Runners

**DOI:** 10.1155/2021/6660304

**Published:** 2021-02-22

**Authors:** Bao Wu, Chang-Cheng Chen, Juan Wang, Xue-Qiang Wang

**Affiliations:** ^1^School of Health and Rehabilitation, Jiangsu Collge of Nursing, Huaian, China; ^2^Department of Sports Rehabilitation, Shanghai University of Sport, Shanghai, China; ^3^Department of Rehabilitation Medicine, Shanghai Shangti Orthopaedic Hospital, Shanghai, China

## Abstract

**Purpose:**

The occurrence of low back pain (LBP) in marathon runners has been poorly understood. This study aimed to describe the risk factors and identify whether these factors can cause LBP in these athletes.

**Methods:**

A self-developed questionnaire was randomly distributed to 850 runners of running a half or a full marathon. Participants responded with the questionnaire focusing on previous training and running conditions after their competitions.

**Results:**

On the basis of the remaining 800 valid questionnaires, the incidence of LBP was 4.50% (*n* = 36). A total of 572 (71.5%) males and 228 (28.5%) females, with an average age range of 33.9 ± 9.0 years, came from different occupations with different physical activity characteristics. However, no significant associations between occupation and runners with LBP (*p* > 0.05) were found. In the final models, risk factors, including warm-up activities (*p*=0.012, OR = 2.617), fatigue (*p* = 0.008, OR = 2.680), running gait posture (*p*=0.041, OR = 2.273), and environmental temperature (*p*=0.020, OR = 6.584), were significantly associated with LBP in marathoners.

**Conclusion:**

Although LBP was uncommon in marathoners, it was linked to the factors such as insufficient warm-up activities, fatigue, poor running gait posture, and uncomfortable environmental temperature. Future studies need to validate these results. Nevertheless, these findings could still be useful for protecting the lower back area of runners clinically.

## 1. Introduction

Marathon running has become an increasingly popular sport in the world in recent years [1]. However, numerous studies have reported the incidence of injuries in long-distance runners during training or competition [2]. To date, the probability of injuries in long-distance athletes is approximately 18.2%–92.4% [3, 4]. In addition, the prevalence of low back pain (LBP) in athletes is 1%–30%, and 10%–15% of all sport injuries were LBP [5, 6]. In marathon runners, previous studies have reported that the incidence rates of LBP were between 4.8% and 10.3% [4, 7, 8]. Many intrinsic and extrinsic risk factors may lead to injuries in athletes, especially in marathon runners [5, 9–11]. Previous studies have reported that intrinsic factors, such as body mass index, body fat percentage, and the circumference of the upper arm, were greatly likely to induce temporary or long-lasting injuries or pain on participants [12–15]. However, no consensus exists on whether age and gender have an effect on injuries [16]. In addition to intrinsic factors, many extrinsic factors (running experience, stretching before exercise, previous injuries, psychological factors, etc.) have been found to induce injuries in long-distance running [16–21].

Although risk factors for running injuries have been discussed extensively in many studies, little literature has documented the specific risk factors of LBP in marathon runners. At present, we only know that the LBP in most athletes is a self-limiting sprain or strain, which gradually develops into a persistent chronic or recurrent symptom and often results in degenerative lumbar disc diseases or spondylotic pressure injuries eventually [5, 22–25]. However, fewer articles have systematically studied what risk factors cause self-limited sprains or strain. In Kasunich's study, lack of muscle strength and inadequate warm-up exercises were considered as the causes of LBP in runners [20]. Malisoux reported that the relationship between the parallel use of different running shoes and running-related injuries in marathon runners with LBP may be positively correlated [8]. Videbaek and Kluitenberg stated that the risk of injuries may be related to the time spent on running in full or half marathon [9, 10].

To prevent LBP in runners, this study aimed to outline the characteristics of risk factors describing LBP in marathon runners, as well as identify whether these factors were related to back pain and injury during a marathon.

## 2. Materials and Methods

### 2.1. Participants

Participants in the 2016 Shanghai International Marathon were invited to answer a self-designed questionnaire. A total of 850 participants running for the full (42.2 km) or half (21.1 km) marathon returned the questionnaire during this event. To decrease bias associated with an unknown magnitude of runners, the inclusion criteria comprised the following: (1) amateur or professional marathoners, (2) ≥18 years old, and (3) runners who can understand the questionnaire. The data with missing basic information or those with incomplete answers were excluded.

### 2.2. Data Collection

Information about LBP and injuries and their related factors obtained from the runners' answers (the remaining 800 valid questionnaires) were collected through a self-developed questionnaire. This questionnaire consisted of basic personal information (such as age, gender, and occupational physical activity characteristics) and contents about risk factors associated with LBP in marathoners. According to our previous study, we confirmed the structural validity (KOM = 0.66) and test-retest reliability (ICC = 0.82) of the questionnaire. Through the questionnaire, we found that the occupations of the participants included managers, business and financial operators, computer programmers, etc. This was not only because of their occupational physical activity characteristics but also because they considered their physical activity to be sedentary. Meanwhile, some participants from professions, such as healthcare instructors, waiters, indoor installation, and maintenance personnel, were self-identified their physical activity as indoor activities. In addition, some professional marathon runners and sports enthusiasts indicated that their physical activities were outdoor activities. There were some student groups and some unidentifiable physical activity occupations in this survey. Therefore, the marathon runners with different occupational physical activity characteristics were simply divided into five groups, namely, sedentary, indoor activity, outdoor activity, student, and other groups. The questionnaire showed the possible factors that may induce LBP in marathoners, including running groups, foot strike patterns, warm-up activities, strength, fatigue, shoe types, running gait posture, previous injuries, temperature factor, site environment, psychological reasons, and other factors. Participants either chose or wrote records in the questionnaire that match their own situation at that condition. Detailed contents are as follows: (1) running groups: full or half marathon; (2) foot strike patterns: forefoot strike (mainly), rear heel strike (mainly), forefoot strike (only), full foot strike, and not clear; (3) warm-up activities: enough or not enough; (4) strength: enough or not enough; (5) fatigue: yes or no; (6) shoe types: slow shaking, controlling, stabilising, sprinting, or not clear; (7) running gait posture: good or bad; (8) previous injuries: yes or no; (9) temperature: comfortable or uncomfortable; (10) site environment: adapted or unadapted; (11) psychology: affected or unaffected; (12) other aspects: yes or no. All questions were responded sequentially and effectively by the runners, and the staff provided an explanation of the question. Especially in terms of running gait posture, we described a good running gait posture as one that holds up its head and looks straight ahead, keeps arm and shoulder swinging naturally, and tries to avoid the distance of per step too wide or too narrow; and the body is moving in the right direction. Given that the marathon runners participating in the survey included amateur athletes and professional marathoners, the content of the risk factors designed in the questionnaire was not only related to professional marathon runners.

### 2.3. Statistical Analysis

The characteristics of the participants were summarised using descriptive statistics (mean ± SD or percentage). In addition, chi-square statistics was used to detect associations between the presence of LBP and participants' characteristics. Univariate logistic regression analysis was used to detect the associations between the presence of LBP and its risk factors (12 factors) in runners (*p* < 0.05) separately. Then, we put the factors which were significant in univariate logistic regression analysis into a stepwise multivariate logistic regression model to determine whether these factors were ultimately associated with the presence of LBP. The independent variables used in the analysis were the risk factors, and the dependent variable was the presence of LBP. Statistical analysis of all results was performed using Statistical Package for Social Sciences software version 22.0.

## 3. Results

### 3.1. Participants

Fifty marathon runners missed basic information or had not completed the questions fully, which were then excluded. Hence, 800 questionnaires proceeded to data analysis. The participants were 572 (71.5%) males and 228 (28.5%) females with average age of 33.9 ± 9.0 years. We found age and gender had no significant correlation with LBP (all *p* > 0.05). In total, approximately 4.50% of all the participants suffered LBP.  Table 1 shows the different occupational physical activity characteristics of age-grouped runners in the marathon. Runners between the ages of 31 and 40 had the highest rate of LBP (2.25%) in all populations. The incidence of LBP in sedentary group was the highest among all population, which accounted for 47.22% of all LBP runners.

### 3.2. Risk Analysis

#### 3.2.1. Univariate Analysis

The univariate logistic regression analysis involved the identification of twelve factors associated with LBP.  Table 2 indicates that six risk factors were significantly related to LBP, particularly the running group of full or half marathon (*p*=0.044), warm-up activities (*p* < 0.001), strength (*p*=0.004), fatigue (*p* < 0.001), running gait posture (*p*=0.003), and environmental temperature (*p*=0.001). Conversely, foot strike patterns, shoe types, previous injury effect, site environment, psychological reasons, and other factors were unrelated to LBP.

#### 3.2.2. Multivariate regression Analysis

Six univariately associated factors (*p* < 0.05) of LBP were utilised in the multivariate logistic regression analysis to develop a risk model.  Table 3 shows that, in the multivariate regression analysis model, warm-up activities (OR = 2.617, 95% CI = 1.237–5.537, *p*=0.012), fatigue (OR = 2.680, 95% CI = 1.292–5.557, *p*=0.008), running gait posture (OR = 2.273, 95% CI = 1.034–4.995, *p*=0.041), and environmental temperature (OR = 6.584, 95% CI = 1.346–32.216, *p*=0.020) were significantly related to LBP in marathoners.

## 4. Discussion

This study assessed the incidence of LBP and estimated the specific risk factors for LBP in marathon runners. The incidence rate was 4.50%, which was slightly lower than the average incidence reported in previous studies [4, 7, 8]. The study also identified that sufficient warm-up activities, no fatigue, good running gait posture, and comfortable environmental temperature are significantly meaningful for the prevention of LBP in marathon runners.

In our study, although a large number of participants were amateur athletes, participants' age and gender were not related to LBP. These results were consistent with other literature [4, 26]. Commonly, occupations are significantly relevant to the incidence of LBP because of the characteristics of occupational physical activities [27, 28]. Occupational physical activities determine the strategies and characteristics of human body use. For example, it is inevitable for long-term sedentary workers to have to flex or bend over for long periods of time. That aggravates the load around the spine, especially the low back, which easily causes the occurrence of LBP. In our research, the occurrence of LBP in the sedentary group was on the top among all the groups that needed to be vigilant.

Warming up prior to exercise or competition is crucial for the attainment of optimum performance [29]. This study found that insufficient warm-up activity was associated with LBP, which was consistent with previous literature reporting that insufficient warm-up activity could cause musculoskeletal injury [26]. The mechanism is that warm-up prior to competition can activate the lower back muscles in advance, promote the stability of the lower back core, and reduce the occurrence of LBP [30]. Consequently, the chance of experiencing passive or active sprain or strain would be reduced. In addition, warm-up activities evoke physiological- and psychological-related effects on the body, thereby greatly enhancing the preparedness for the subsequent task [31–34]. This preparation also reduces the risk of injuries in some way. However, warm-up activities can also bring the negative effect of performance, which may induce fatigue by excessive tension and lack of adequate recovery before a competition [35, 36]. Therefore, warm-up activities should be appropriately balanced before training or competition.

Fatigue is inevitable in all athletes and is an essential aspect when exploring the full-performance capabilities of runners [37]. In our study, we found fatigue was one of the risk factors that was associated with LBP in marathon runners. The mechanism is that excessive use of the body function and heavily repetitive physical work on the waist region when running can aggravate the waist load, leading to provoking LBP [38, 39]. Especially when runners were tired, they tended to lose control of their bodies, especially their waist movements. At that condition, runners cannot be able to take advantage of the redundancy of the musculoskeletal system to adapt to their movement strategies, thereby accumulating injury tissues [40]. Meanwhile, fatigue leads to an imbalance of immune suppression, impaired reproductive function, and changes in physical self-perception of the body [41]. All these effects will lead to an abnormal endogenous body regulation that may cause the occurrence of LBP indirectly. Commonly, amateur marathon runners without adequate professional training or exercise were most likely to feel tired and became a high risk of population with LBP [40, 42, 43], because runners lost control of lumbar movement and decreased the structured lumbar movement variability after the fatigue, which was associated with increased LBP.

Distance running performance needs running gait posture to collaborate, including stride parameters, lower limb angles, vertical displacement of the body, changes in horizontal velocity during ground contact, and trunk and pelvis orientation [1, 44]. In our research, we found bad running gait posture was related to LBP in marathon runners. The mechanism is that improper running gait posture can cause changes in back-surrounding muscles, spine, and pelvis biomechanics. Abnormal biomechanics changes naturally cause the lower back injuries [44]. However, considering that running is a relatively independent movement with multiple degrees of freedom, individual runners perform forward locomotion by using multifarious strategies with evidence for large interindividual shifts in stride patterns and lower limb kinematics [45, 46]. Therefore, further research is needed in the future to confirm the extent to which running gait posture affects LBP with different exercise strategies.

In our study, we found comfortable environmental temperature was associated with the reduction of the occurrence of lower back injuries. Previous studies have reported that changes in ambient temperature on the performance of exercise could affect the central system to regulate our body to adapt to the environment [47]. We also knew ambient temperature could cause changes in the cardiovascular blood supply to the working muscles [48]. Therefore, the uncomfortable ambient temperature when running marathon affected the normal state of the lower back through a combination of the nervous and cardiovascular system, which may be the mechanism of LBP. However, the result needs to be confirmed in the future with additional experiments to be conducted. Nonetheless, strategies to prevent LBP for marathoners should consider appropriate competition for the race based on environmental temperature [49, 50]. Athletes should acclimate to the running environment, especially the temperature if possible.

When analyzing other factors, this study did not find that the shoe factor was a risk factor of LBP occurrence. Although studies have reported that previous injuries might be the cause of injuries, previous injuries were not a significant factor in this survey. Furthermore, different running groups had nothing to do with the occurrence of LBP in marathon runners. However, the results of the present study should still be carefully considered.

The limitation of this study was the limited sample size of the LBP in the half or full marathon runners. Only 36 runners of the total 800 responders reported their back pain compared with the remaining 764 participants with low back health. This result indicates the need to increase the data to supplement analyses related to LBP in marathon runners and analyze whether the risk factors about back pain are trustworthy with large sample experiments. Another limitation was the content of the design associated with the occurrence of LBP displayed in the questionnaire. Not all aspects of types of lower back injuries and pain in marathon, such as LBP-related pelvic movement and pain symptoms, were included. Also, the frequency of training per day or total training time in male and female marathon runners and three general types of LBP by causes (mechanical back pain, nonmechanical back pain, and referred pain from internal organs) were not fully considered. And the lost characteristics of BMI were not included in the analysis. Meanwhile, given that this study was cross-sectional with an exploratory nature, determining the continuous cause-effect relationship between LBP and its risk factors in marathon was difficult. Hence, further research is needed to obtain a complete investigation on the occurrence of LBP in marathon runners, including timely feedback on the physical condition before the marathon and a follow-up survey after the competition, to determine further the factors that cause LBP in marathon runners.

## 5. Conclusions

This self-reported study revealed a 4.50% prevalence of running-related injuries of LBP in marathon runners. Factors, including warm-up activities, fatigue, running gait posture, and environmental temperature, were related to an increased risk of LBP in general. Therefore, if planning to prepare a marathon, sufficient strength training and adapting to environmental temperature in advance are highly essential for runners to prevent LBP. Also, an appropriate amount of regular warm-up activities to mobilise the entire body beforehand is a good choice to enhance the health of the lower back area. Meanwhile, the results may be a combination of special conditions that still need further studies for confirmation.

## Figures and Tables

**Table 1:** Occupational physical activity characteristics of different age groups for runners.

Age groups (%)	Conditions	Sedentary groups	Indoor activity groups	Outdoor activity groups	Student groups	Other groups	Total	Incidence (LBP) (%)
		Number	Number	Number	Number	Number	Number	
18–20	Non-LBP	6	3	5	8	0	22	0.125
	LBP	0	0	0	1	0	1	
	Total	6	3	5	9	0	23	

21–30	Non-LBP	163	25	55	29	15	287	1.75
	LBP	5	1	6	1	1	14	
	Total	168	26	61	30	16	301	

31–40	Non-LBP	204	15	59	0	21	299	2.25
	LBP	10	3	4	0	1	18	
	Total	214	18	63	0	22	317	

41–50	Non-LBP	69	7	22	1	18	117	0.25
	LBP	1	0	1	0	0	2	
	Total	70	7	23	1	18	119	

51–60	Non-LBP	22	1	4	0	5	32	0.125
	LBP	1	0	0	0	0	1	
	Total	23	1	4	0	5	33	

≥61	Non-LBP	0	2	3	0	2	7	0
	LBP	0	0	0	0	0	0	
	Total	0	2	3	0	2	7	

Total (LBP)		17	4	11	2	2	36	4.50

Non-LBP: non-low back pain; LBP: low back pain.

**Table 2:** Summary of risk factors for LBP in marathon runners.

Risk factors	Non-LBP *N* = 764	LBP (%) *N* = 36	Total (%) *N* = 800	*p* value
	Number (%)	Number (%)	Number (%)	
*Running groups*				
Full marathon	314 (41.1)	21 (58.3)	335 (41.9)	0.044^*∗*^
Half marathon	450 (58.9)	15 (41.7)	465 (58.1)	

*Foot strike patterns*				
Forefoot (main)	396 (51.8)	21 (58.3)	417 (52.2)	0.139
Rear heel (main)	268 (35.1)	13 (36.1)	281 (35.1)	
Forefoot (only)	15 (2.0)	1 (2.8)	16 (2.0)	
Full foot	41 (5.4)	0 (0)	41 (5.1)	
Not clear	44 (5.7)	1 (2.8)	45 (5.6)	

*Warm-up activities*				
Enough	648 (84.8)	21 (58.3)	669 (83.6)	<0.001^*∗*^
Not enough	116 (15.2)	15 (41.7)	131 (16.4)	

*Strength*				
Enough	636 (83.2)	23 (63.9)	659 (82.4)	0.004^*∗*^
Not enough	128 (16.8)	13 (36.1)	141 (17.6)	

*Fatigue*				
Yes	123 (16.1)	15 (41.7)	138 (17.3)	<0.001^*∗*^
No	641 (83.9)	21 (58.3)	662 (82.7)	

*Shoe types*				
Slow shaking	365 (47.8)	17 (47.2)	382 (47.8)	0.664
Controlling	64 (8.4)	4 (11.1)	68 (8.5)	
Stabilising	217 (28.4)	12 (33.3)	229 (28.6)	
Sprinting	24 (3.1)	1 (2.8)	25 (3.1)	
Not clear	94 (12.3)	2 (5.6)	96 (12.0)	

*Running gait posture*				
Good	669 (87.6)	25 (69.4)	694 (86.7)	0.003^*∗*^
Bad	95 (12.4)	11 (30.6)	106 (13.3)	

*Previous injuries*				
Yes	49 (6.4)	3 (8.3)	52 (6.5)	0.649
No	715 (93.6)	33 (91.7)	748 (93.5)	

*Temperature*				
Comfortable	758 (99.2)	33 (91.7)	791 (98.9)	0.001^*∗*^
Uncomfortable	6 (0.8)	3 (8.3)	9 (1.1)	

*Site environment*				
Adapted	748 (97.9)	34 (94.4)	782 (97.7)	0.189
Unadapted	16 (2.1)	2 (5.6)	18 (2.3)	

*Psychology*				
Affected	7 (0.9)	1 (2.8)	8 (1.0)	0.298
Unaffected	757 (99.1)	35 (97.2)	792 (99.0)	

*Other aspects*				
Yes	30 (3.9)	1 (2.8)	31 (3.8)	0.728
No	734 (96.1)	35 (97.2)	769 (96.2)	

Non-LBP: non-low back pain; LBP: low back pain. ^*∗*^*p* < 0.05.

**Table 3:** Multivariate regression model (forward stepwise) for LBP in marathoners.

Risk factors	OR	95% CI	*p* value
Warm-up activities:	2.617	1.237–5.537	0.012^*∗*^
Enough vs. not enough			

Fatigue:	2.680	1.292–5.557	0.008^*∗*^
Yes vs. no			

Running gait posture:	2.273	1.034–4.995	0.041^*∗*^
Good vs. bad			

Temperature	6.584	1.346–32.216	0.020^*∗*^
Comfortable vs. uncomfortable			

Non-LBP: non-low back pain; LBP: low back pain. ^*∗*^*p* < 0.05.

## Data Availability

The data used to support the findings of this study are available from the corresponding author upon request.
